# Case Report: Uncommon cause of limp in the 21^st^ century

**DOI:** 10.3389/fendo.2022.968015

**Published:** 2022-08-01

**Authors:** Stephanie Thiemann, Valeria Cimorelli, Nadia M. Bajwa

**Affiliations:** ^1^ Department of Women, Children, and Adolescents, Service of General Pediatrics, Geneva University Hospitals, Geneva, Switzerland; ^2^ Unit of Development and Research in Medical Education, University of Geneva Faculty of Medicine, Geneva, Switzerland

**Keywords:** children, malnutrition, vitamin C, ascorbic acid, scurvy, weakness, limp

## Abstract

Scurvy results from a deficiency of ascorbic acid. This disease first appeared in children during the 19th century with the emergence of new dietary habits; in particular, heating milk that leads to a loss of ascorbic acid. Even though scurvy has become a rare condition in western countries, many cases are still reported in pediatric patients, especially in those who lack proper nutrition due to neurological or psychiatric illnesses. Symptoms include bleeding and swollen gums, loosening of teeth, bone abnormalities, arthralgia, delayed wound healing, anemia, petechiae, and purpura. Bone lesions are mainly irregularities of long bones metaphyses. We report the case of a five-year-old boy who presented with arthralgia and limb deformation (genu valgum). The patient was investigated for vitamin deficiencies to exclude rickets. The radiologic investigations revealed metaphyseal signs compatible with scurvy. During the hospitalization, the patient was observed to have abnormal eating patterns and the scurvy was attributed to malnutrition. Although the occurrence of scurvy is rare, it remains essential to detect this disease in children at risk of developing vitamin deficiencies. Without targeted treatment, the complications of scurvy can be serious and potentially fatal.

## Introduction

The diagnosis of scurvy is oftentimes one that takes place in clinical textbooks rather than in a clinical consultation. Vitamin C (ascorbic acid) is an exogenous, water-soluble vitamin that is required for the biosynthesis of collagen, L-carnitine, and certain neurotransmitters. It is also an important antioxidant. Severe vitamin C deficiency leads to abnormal collagen formation in blood vessels, skin, and tissues. Symptoms classically include bleeding, painful gums in the malnourished. Early constitutional symptoms may include abdominal pain, anorexia, weight loss, fever, and fatigue (1). Late onset symptoms are likely to include musculoskeletal manifestations such as weakness, inability to bear weight, limp, arthralgia, and myalgia (1). However, this illness may mimic a variety of rheumatological, orthopedic, neurological, and hematological illnesses. Sir James Lind in 1753 was the first to report the consumption of citrus fruits as a method to prevent scurvy (2). In modern times, patients presenting with scurvy often have comorbid prematurity, psychiatric, or neurological disorders that may lead to malnutrition (3). A lack of screening of patients with relevant comorbidities may be contributing to the resurgence of this illness (3). Studies on the prevalence of vitamin C deficiency in the general population in the USA have shown a prevalence of 7.1%; cases tend to occur more often in patients with a low socio-economic status and are usually asymptomatic (4). In developing countries, such as Mexico, vitamin C deficiency in children may reach up to 30% (5). We present the case of a child with a limp who was investigated for rheumatological, orthopedic, and oncologic illnesses until a nutrition history raised the suspicion of scurvy. Clinicians must maintain a high index of suspicion to make this rare diagnosis.

## Case description

**Figure 1:**
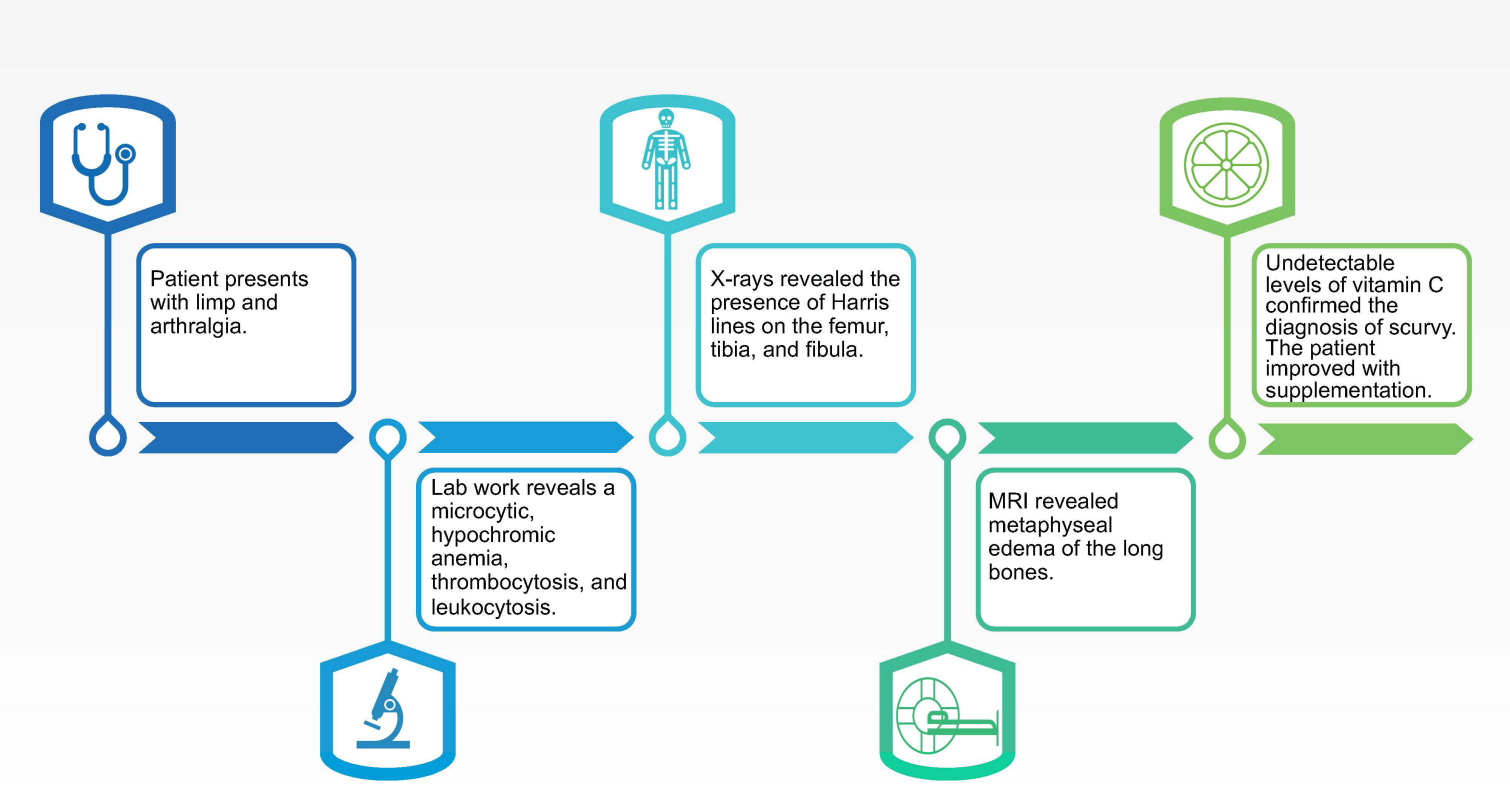
Timeline of relevant events in the patient history.

A 5-year-old boy consulted his pediatrician for refusal to walk and lower limb pain. The clinical picture began eight months prior with intermittent joint pain localized to the ankles, hips, back, and knees. There was no history of trauma, and the patient showed no signs of infection. His medical history was notable for prematurity (34 2/7 weeks gestational age) associated with a twin pregnancy. No neonatal complications were documented. The patient was noted to be a toe walker until the age of three. On physical evaluation, osteoarticular infection seemed unlikely as the patient had no fever or joint inflammation. Although it could not be formally ruled out, genetic muscle disorders seemed less likely given the age of onset of symptoms and there was a predominance of pain rather than muscle weakness. The joint pain was initially attributed to be growing pains. The clinical picture progressively worsened with the pain becoming disabling and preventing the child to walk by himself leading to school absenteeism. The family noted a progressive knee deformation described as genu valgum. A timeline of the patient’s course is available in **Figure 1**.

Blood tests revealed a negative CRP and a non-regenerative microcytic hypochromic anemia associated with a discrete thrombocytosis and leukocytosis. Because of the knee complaints, the pediatrician completed lab work with an assessment of calcium and phosphorus metabolism (serum calcium was normal at 2.43 mmol/L, phosphorus was normal at 1.67 mmol/L, and alkaline phosphatase was normal at 226 U/L) to rule out rickets. The results showed a normal 25-OH vitamin D level (25-OH vitamin D level was 66nmol/L, reference range above 50 nmol/l). The renal function, as well as CK levels and hepatic enzymes were normal, excluding a muscular condition. Blood tests confirmed that the microcytic hypochromic anemia was secondary to an iron deficiency. Iron levels were low at 4μmol/L, the index of saturation was low at 0.05, and the ferritin level was low at 10μg/L. Iron supplementation and vitamin D supplementation were prescribed. Platelets remained slightly elevated due to the iron deficiency. All inflammatory markers remained normal (white blood cell count, CRP, ESR). Muscular markers were normal (alanine aminotransferase, aspartate aminotransferase, creatine kinase, aldolase, lactate dehydrogenase, antinuclear antibody, antibodies specific for dermatomyositis) making the diagnosis of dermatomyositis less likely.

**Figure 2:**
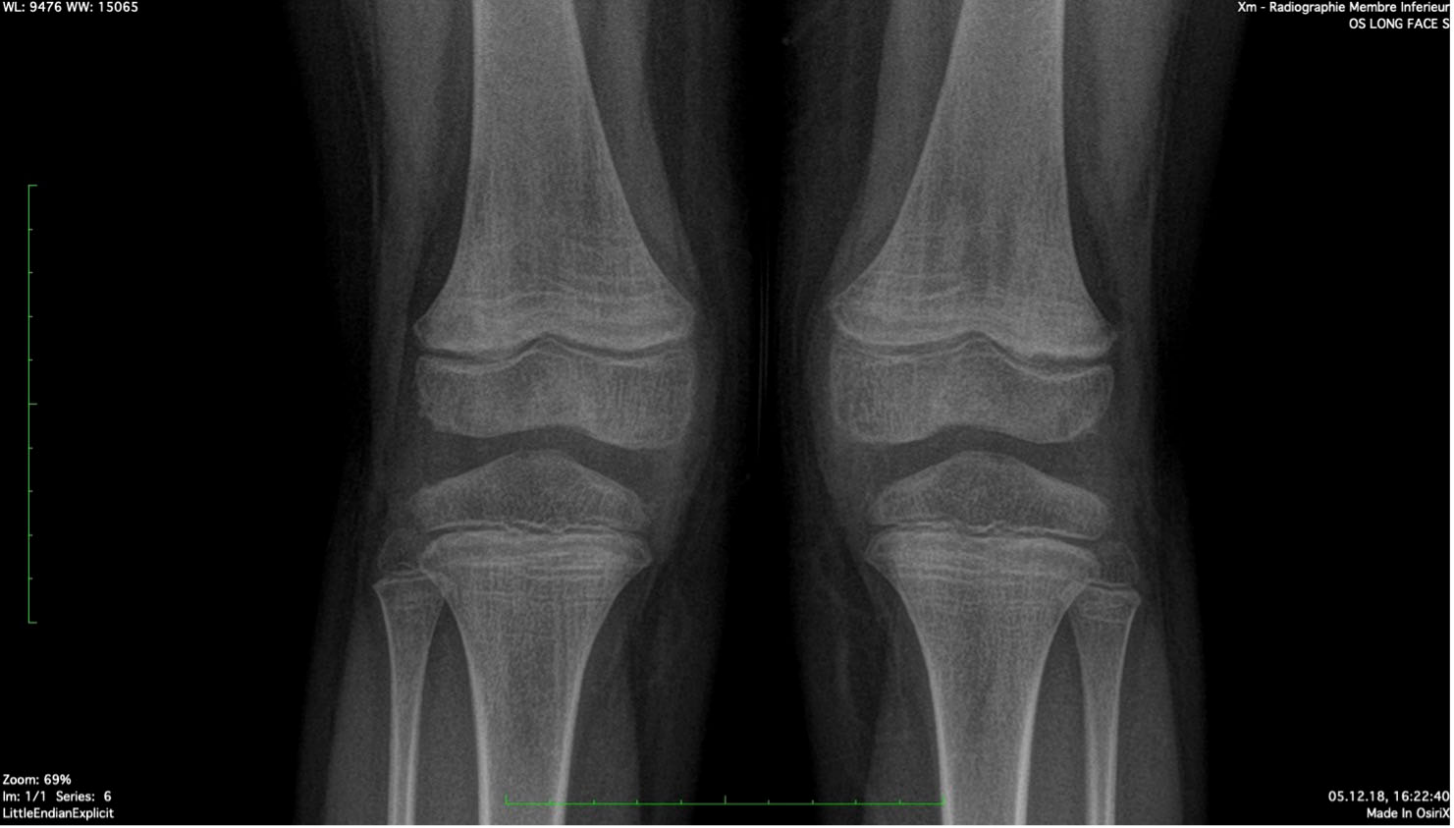
Harris Lines are visible as radiopaque transverse lines located in the metaphyses of the long bones (femur and tibia in this picture). These lines are the result of growth arrest periods, but can also be seen in cases of heavy metal intoxication.

The patient was referred to an orthopedic surgeon to complete the investigations. A congenital condition was excluded with the clinical exam. A lower limb X-ray excluded neoplastic causes and revealed the presence of Harris lines in the metaphyses of the femur, tibia, and fibula; see **Figure 2**. Harris lines on the X ray and edema on metaphyseal bones can reflect growth arrest lines. These are associated with malnutrition and certain types of poisoning (e.g., heavy metals). Genu valgum was also noticed. Notably, there was no evidence of osteopenia with cortical thinning nor signs of abnormal calcification or periosteal elevation.

The patient was then referred to the pediatric department for investigation. Upon admission, the vital signs were normal. The patient’s weight was 33.3 kg (>99th percentile), height 124.7 cm (>99th percentile), and the body mass index was 21.6 kg/m2 (>99th percentile). The obesity was primarily truncal. The child was pale but not ill-appearing. The oral cavity was normal as well as the cardiovascular, respiratory, and abdominal evaluation. Physical examination of the lower limbs showed bilateral genu valgum without any other deformity. The skin and hair did not reveal any changes or signs of local inflammation (hemorrhages, purpura, or corkscrew hair deformity). There was no evidence of soft tissue swelling or muscle atrophy. Bony palpation was remarkable only for pain on the right lateral malleolus. All joints were free of limitation. Foot position was remarkable for bilateral external rotation. Walking gait examination showed slow motion of the trunk, decreased stride length, and slow stride frequency. Knees were slightly flexed. The patient was able to walk without help, with a limp caused by the pain. Heel walking and toe walking were impossible because of the pain. There was no weakness of the flexor plantar or extensor muscles. Osteotendinous reflexes were normal. An ophthalmologic exam was not performed.

**Figure 3:**
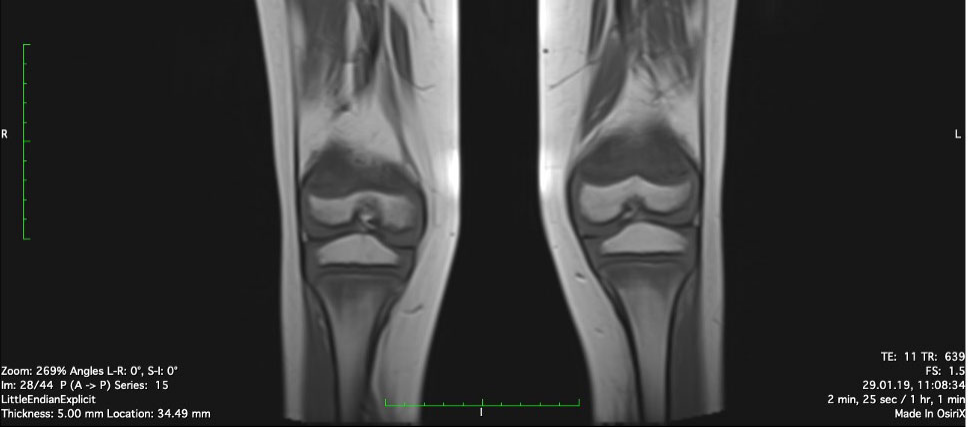
Edema of metaphyseal areas is visible in the T1 sequence as hypointense signals in the affected areas.

**Figure 4:**
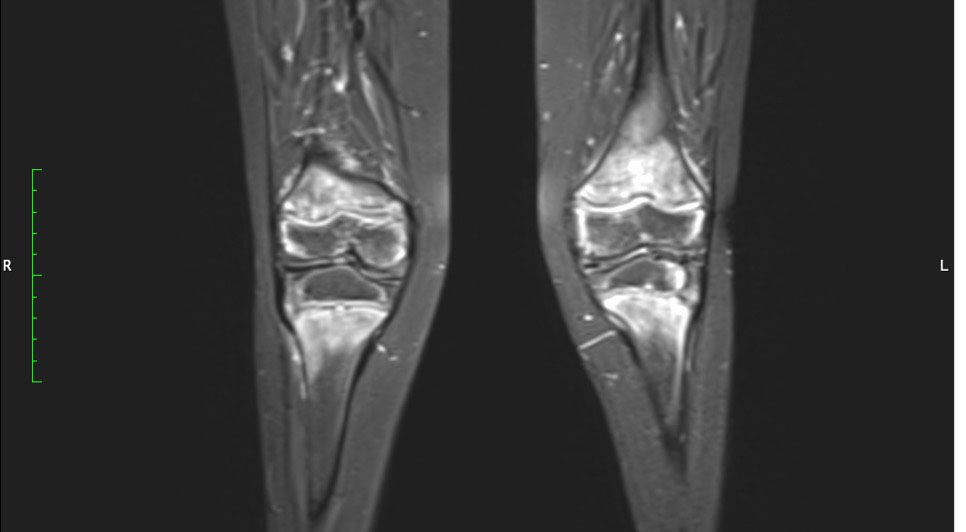
Hyperintense signals are present in the T2 MRI sequence in metaphyseal areas, compatible with metaphyseal edema of long bones.

Physiotherapy was started in order to improve the muscular function. Taking into account the patient’s chronic multifocal pain and limited ability to function, we suspected a chronic recurrent multifocal osteomyelitis (CRMO). Whole-body MRI (WBMRI) revealed metaphyseal edema of the long bones (elbows excluded), pubic arches, and sacroiliac joints (**Figures 3**, **4**). The involvement was bilateral, symmetrical, and without epiphyseal or joint involvement. The spleen was enlarged (most likely in relation to the iron deficiency anemia). Muscles showed no abnormalities and definitively excluded dermatomyositis. The spine was normal. The WBMRI was able to exclude a CRMO.

Signal abnormalities of metaphyseal areas in the MRI were highlighted by our radiologists and were suggestive for a metabolic disease or a hematological condition (**Figure 4**). A bone marrow biopsy and flow cytometry were then performed and ruled out leukemia or other infiltrative processes.

The combination of Harris lines described in the X ray and metaphyseal edema in the WBMRI, widened the differential diagnosis to vitamin deficiencies or heavy metal intoxications. The dietary history revealed a diet exclusively based on cheese and carbohydrates and lacking of fruits and vegetables. The patient came from a family that belongs to a high socio-economic level with access to nutrient rich foods. However, the parents related that since the diversification of foods in infancy their son was resistant to eating fruits and vegetables which caused them to rely solely upon carbohydrates and cheese. We completed lab work with vitamin deficiency and lead poisoning screening which revealed folate deficiency and an undetectable vitamin C level. This confirmed the diagnosis of juvenile scurvy. Folate and vitamin C oral supplementation (1 mg/d and 500mg 1x/day, respectively) were started, and we observed a significant decrease of pain and the child resumed walking. In addition to vitamin supplementation, significant patient and parent education were needed in order to educate the family about the importance of a balanced diet and to find suitable strategies for integrating vitamin C rich foods into the patient’s restrictive diet. Long-term follow-up is currently ongoing to ensure that the patient’s diet restrictions are not related to an eating disorder and that other vitamin deficiencies do not occur in the future. As the patient remained asymptomatic, follow-up imaging was not deemed necessary.

## Discussion

Scurvy is an old disease that became famous in the era of the Renaissance, which witnessed an outbreak among sailors as a consequence of a prolonged lack of vitamin C during overseas expeditions. Infantile scurvy appeared later in the 19 century with the emergence of new alimentary habits, in particular, heating milk that lead to a loss of many vitamins (6, 7).

Scurvy results from a deficiency of ascorbic acid, also known as vitamin C, found almost exclusively in fruits and vegetables. Human cells have a pool of ascorbic acid estimated around 1500-2500 mg. Vitamin C is a water-soluble vitamin that is effectively absorbed from the gastrointestinal tract and excess vitamin C is excreted in the urine. Vitamin C has a half-life of 10-20 days, which explains why scurvy only appears if there is a prolonged deficiency in the diet (8). The recommended dietary allowance (RDA) of vitamin C is approximatively 95-110mg/day for adults (9). For children the RDA is age dependent: 40 mg/day for 0 to 6 months old, 50 mg/day for 6 to 12 months old, 15mg/day for 1 to 3 years old, 25 mg/day for 4 to 8 years old, 45 mg/day for 9 to 13 years old, and 65 to 75 mg/day for 14 to 18 years old (10). During breastfeeding, intake is covered by maternal milk. Rich sources of vitamin C include citrus fruits such as oranges, lemons, and limes as well as vegetables such as broccoli, peppers, and potatoes.

Vitamin C plays many roles in the human body. Among them, vitamin C is an antioxidant and a cofactor for many enzymes such as those required in collagen synthesis, fatty acid transport, neurotransmitter synthesis, prostaglandin metabolism, and nitric oxide synthesis (9). Unlike other living organisms, our body cannot synthesize ascorbic acid due to a lack of the gene for the l-gulonolactone oxidase enzyme which converts glucose into ascorbic acid which is why an imbalanced diet can lead to scurvy (11).

Initial symptoms of scurvy are mostly constitutional and may go unnoticed. Arthralgia and muscular pain are present in more than 80% of cases and explained by the weakness of several structures such as ligaments, bones and muscle fibers (8, 12). Pain is also directly caused by synovial bleeding, subperiostal hemorrhages, and microfractures (12). Bleeding and delayed wound healing are explained by capillary fragility. Swollen gums and loose teeth are tardive symptoms.

Laboratory findings are unspecific and globally indicate the patient’s malnutrition. Blood samples may reveal a hypochromic anemia, iron deficiency, folic acid deficiency, and hypoalbuminemia. Vitamin C levels are necessary to make the diagnosis as well as to document recovery after vitamin C substitution. A plasma ascorbate concentration of <0.2 mg/dL indicates a deficiency.

Several radiological findings may be indicative of scurvy. In general, there is the presence of osteopenia with cortical thinning. Frankel’s line which is described as increased density and widening of the zone of provisional calcification may also be present (13). The Trümmerfeld zone is a radiolucent line parallel to Frankel’s line (13). Pelkan’s spur reflects calcification and periosteal elevation beyond the metaphysis and Wimberger’s ring sign denotes a circular calcification around an osteopenic epiphysis (13). MRI findings classically consist of diffuse multifocal decreased signal on T1-weighted imaging and increased signal on T2-weighted imaging most often within the metaphyses (13). Bone marrow enhancement as well as adjacent periosteal elevation and soft tissue abnormalities may also be present (13).

The differential diagnosis for chronic nontraumatic limb pain associated with limp in a school-aged child should include infectious etiologies (septic arthritis, osteomyelitis, and spondylodiscitis), neoplastic causes (osteosarcoma, Ewing’s sarcoma, leukemia, lymphoma, spinal cord tumor, metastatic neuroblastoma), neurological conditions (hereditary sensory motor neuropathies, complex regional pain syndrome), inflammatory diseases (postinfectious reactive arthritis, juvenile idiopathic arthritis, acute rheumatic fever), congenital conditions (dysplasia of the hip, limb-length discrepancy) and orthopedic disorders (Legg-Calve-Perthes) (14).

Our case is an example of the importance of taking a detailed history including a nutritional history. Had we included nutritional deficiencies in our initial differential diagnosis, we would have been able to circumvent unnecessary lab testing, imagery, invasive procedures, and would have been able to treat the patient more quickly.

Treatment of scurvy involves the supplementation of vitamin C. Multiple treatment regimens exist. Patients may be supplemented intravenously (IV) or orally depending on the severity of symptoms. Typically, infants and children may receive 100 to 300 mg orally per day or 1g IV for 1 week (1, 11). Patients are then supplemented with 100 mg daily for 1 to 3 months. Constitutional symptoms generally resolve within 24 hours while cutaneous and musculoskeletal symptoms may take several weeks to improve.

## Conclusion

Scurvy has become a challenging diagnosis in developed countries because of the rarity of the condition. However, it is a serious condition that needs to be treated without delay, otherwise it can lead to a fatal outcome. Despite unspecific symptoms, it is a multisystemic disease. Prevention remains the central pillar of medical management of this disease. Among pediatric patients, vitamin deficiencies are common and should be screened for and detected as early as possible, especially in children at risk. Dietary and development history should always be part of the child’s overall assessment. Children with neurological or psychiatric conditions which can cause them to have improper nutrition are the most likely to be reported in the literature.

## Data availability statement

The datasets for this article are not publicly available due to concerns regarding participant/patient anonymity. Requests to access the datasets should be directed to the corresponding author.

## Ethics statement

Ethical review and approval were not required for the study on human participants in accordance with the local legislation and institutional requirements. Written informed consent was obtained from the minor(s)’ legal guardian/next of kin for the publication of any potentially identifiable images or data included in this article.

## Author contributions

ST, VC, and NB all participated in the drafting of the manuscript. ST, VC, and NB were also responsible for the care of the patient. All authors revised the manuscript and approved the content before submission.

## Funding

Open access funding was provided by the University of Geneva.

## Acknowledgments

We thank the patient and his family for participating in this study.

## Conflict of interest

The authors declare that the research was conducted in the absence of any commercial or financial relationships that could be construed as a potential conflict of interest.

## Publisher’s note

All claims expressed in this article are solely those of the authors and do not necessarily represent those of their affiliated organizations, or those of the publisher, the editors and the reviewers. Any product that may be evaluated in this article, or claim that may be made by its manufacturer, is not guaranteed or endorsed by the publisher.
