# Erratum: Case Report: Uncommon cause of limp in the 21st century

**DOI:** 10.3389/fendo.2023.1178917

**Published:** 2023-03-16

**Authors:** Frontiers Production Office

**Affiliations:** Frontiers Media SA, Lausanne, Switzerland

**Keywords:** children, malnutrition, vitamin C, ascorbic acid, scurvy, weakness, limp

An omission to the funding section of the original article was made in error. The following sentence has been added: “Open access funding was provided by the University of Geneva”.

The original version of this article has been updated.

